# CEMIP (KIAA1199) regulates inflammation, hyperplasia and fibrosis in osteoarthritis synovial membrane

**DOI:** 10.1007/s00018-022-04282-6

**Published:** 2022-04-27

**Authors:** Céline Deroyer, Christophe Poulet, Geneviève Paulissen, Federica Ciregia, Olivier Malaise, Zelda Plener, Gaël Cobraiville, Christophe Daniel, Philippe Gillet, Michel G. Malaise, Dominique de Seny

**Affiliations:** 1 Laboratory of Rheumatology, GIGA-Research, CHULiège, ULiège, 4000 Liège, Belgium; 2 Department of Orthopaedic Surgery, CHULiège, 4000 Liège, Belgium

**Keywords:** CEMIP, KIAA1199, Hybid, Fibrosis, Inflammation, Osteoarthritis, Synovitis

## Abstract

Osteoarthritis (OA) synovial membrane is mainly characterized by low-grade inflammation, hyperplasia with increased cell proliferation and fibrosis. We previously underscored a critical role for CEMIP in fibrosis of OA cartilage. However, its role in OA synovial membrane remains unknown. An in vitro model with fibroblast-like synoviocytes from OA patients and an in vivo model with collagenase-induced OA mice were used to evaluate CEMIP-silencing effects on inflammation, hyperplasia and fibrosis. Our results showed that i. CEMIP expression was increased in human and mouse inflamed synovial membrane; ii. CEMIP regulated the inflammatory response pathway and inflammatory cytokines production in vitro and in vivo; iii. CEMIP induced epithelial to mesenchymal transition pathway and fibrotic markers in vitro and in vivo*;* iv. CEMIP increased cell proliferation and synovial hyperplasia; v. CEMIP expression was increased by inflammatory cytokines and by TGF-β signaling; vi. anti-fibrotic drugs decreased CEMIP expression. All these findings highlighted the central role of CEMIP in OA synovial membrane development and underscored that targeting CEMIP could be a new therapeutic approach.

**Supplementary Information:**

The online version contains supplementary material available at 10.1007/s00018-022-04282-6.

## Introduction

Osteoarthritis (OA) is the most common chronic joint disease affecting one or several joints, especially knee, hip, hand or spine. Several risk factors are identified like age, gender or BMI, but the precise origins are still unknown [1]. Currently, medical treatments only allow pain reduction and remain ineffective against OA joint tissue degradation. In severe cases, prosthetic surgery can be implemented for the knee or hip [2].

The OA joint displays cartilage degradation, subchondral bone sclerosis, synovitis and osteophytes formation. OA synovitis is characterized by hyperplasia, stroma vascularization and inflammatory cell infiltration with pro-inflammatory cytokines production and fibrosis [3]. OA was for long considered as a degenerative disease, but it is increasingly admitted that inflammation of synovial membrane is a precocious and an important feature in OA development. Moreover, recent studies have indicated that synovitis could reflect pain, joint dysfunction and disease severity [4, 5]. Synovitis could result from the degradation of cartilage and other joint tissues leading to the release of pro-inflammatory cytokines (i.e., IL1-β, TNF-α) and chemokines [5].

Recently, we underscored the inflammatory gradient existing in synovial membrane from OA, chronic pyrophosphate arthropathy (CPPA), and rheumatoid arthritis (RA) patients, and we showed that synovitis was characterized by the overexpression of endoplasmic reticulum stress proteins [6]. Synovial membrane fibrosis can result from chronic inflammation and is depicted by excessive production of extracellular matrix components and αSMA expression [5, 7, 8]. TGF-β1 signaling is considered as the main pathway responsible for fibrosis in OA [7]. In this context, we recently showed that vitronectin (VTN) fragment (381–397 a.a.), through its interaction with α_V_β_6_, could activate latent TGF-β1 and induce fibrosis in OA fibroblast-like synoviocytes (FLS) [9]. While inflammation is observed in the early stage of OA, fibrosis is reported in the late stage [10, 11]. Inflammation of synovial membrane induces hyperplasia and cartilage degradation, leading to joint pain and is an important trigger of fibrosis [12]. Fibroblast proliferation and collagen deposition due to fibrosis induce the development of a thick and rigid membrane that contributes to joint pain and stiffness [13, 14]. In addition to the synovial membrane, fibrosis is also encountered in OA cartilage [15–17]. Previously, we highlighted that CEMIP, through the activation of TGF-β signaling, induces fibrosis in OA cartilage [16].

CEMIP is a protein of emerging interest in arthritis diseases. Indeed, several recent studies have highlighted its potential role in RA and in OA development. In RA, CEMIP expression is increased in FLS from RA patients compared to healthy controls [18]. Moreover, CEMIP overexpression is also found in serum, synovial fluid and synovial tissue of RA patients and correlates with disease severity [19]. CEMIP expression is increased in synoviocytes of OA patients, compared to healthy controls, but remains lower compared to those of RA patients [18]. Moreover, CEMIP is overexpressed in OA cartilage compared to normal cartilage and this overexpression is correlated with hyaluronic acid (HA) loss and disease severity [16, 20].

Some lines of explanation for this overexpression in arthritis tissues have been recently emphasized and correlated to the presence of pro-inflammatory cytokines in OA joint cavity. In OA chondrocytes, CEMIP expression is induced by TNF-α and IL-1β, the latter being under the control of ERK activation and NF-κB nuclear translocation [20, 21]. In OA synoviocytes and in Crohn’s disease fibroblasts, IL-6 significantly up-regulated CEMIP level [22, 23]. Finally, in human skin fibroblasts, histamine increased CEMIP expression [18].

In addition to its overexpression in arthritis tissues, it is increasingly apparent that CEMIP could play a critical role in pathology development. Indeed, CEMIP plays a key role in the catabolism of HA. It induces high molecular weight HA degradation into low molecular weight HA, leading to inflammation and angiogenesis [18, 20, 22]. Moreover, it has been demonstrated that CEMIP regulates pathways involved in OA cartilage such as Wnt/β-catenin and TGF-β signaling [16, 24].

We recently showed that CEMIP induced chondrocytes trans-dedifferentiation into chondro-myofibroblasts expressing fibrotic markers and therefore contributed to OA cartilage progression [16]. In the present study, we investigated the pathological role of CEMIP in the OA synovial membrane, and specifically in the inflammation, hyperplasia and fibrosis process. The in vitro model allowed to investigate each feature separately with TNF-α or TGF-β stimulations, whereas the in vivo OA model allowed to have a global view on the contribution of CEMIP in OA development. In addition, based on high-throughput RNA-sequencing analysis of Cemip depleted cells, we proposed mechanistic explanations for this regulation and investigated how CEMIP is regulated in OA FLS.

## Results

### CEMIP expression in inflamed synovial membrane

**Fig. 1:**
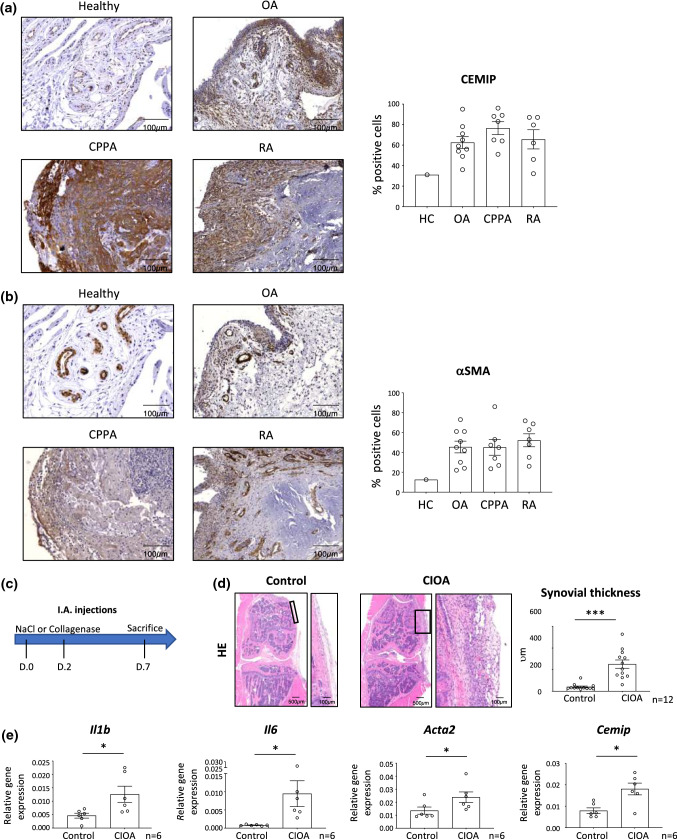
CEMIP and αSMA expression in healthy and inflamed synovial membrane Representative histological pictures of human synovial membrane sections from healthy (*n* = 1), OA (*n* = 9), CPPA (*n* = 7) and RA (*n* = 6) joints. Synovial sections were stained with anti- CEMIP antibody (**a**) or anti-αSMA antibody (**b**). CEMIP and αSMA expressions were quantified and are expressed in percentage of positive stained cells. Schematic representation of I.A collagenase injections in mice (**c**). Representative histological pictures of mouse synovial membrane sections from healthy joints (NaCl) or inflamed OA joints (CIOA) (*n* = 12). Synovial thickness was measured from hematoxylin–eosin coloration and expressed in μm (**d**). RT-qPCR analysis of *IL1B*, *IL6, ACTA2* and *CEMIP* genes in synovial membrane of CIOA and control knees (NaCl) (*n* = 6) (**e**). Data are expressed as mean with SEM. Parametric paired *t* tests (for values that pass normality test) or non-parametric Wilcoxon tests (for values that did not pass normality test) were applied. *p < 0.05 and ***p < 0.001

CEMIP expression was evaluated in human synovial biopsies from healthy control (HC) (*n* = 1), OA (*n* = 9), CPPA (*n* = 7) and RA (*n* = 6) patients (Fig. 1a). The mean expression of CEMIP in synovial membranes was 31% in HC, 62.5% (36–95) in OA, 76.5% (51–96) in CPPA and 65.6% (32–87) in RA. In the healthy synovial membrane, CEMIP was mainly expressed in blood vessels and slightly in subintima. In OA biopsies, in addition to blood vessels, CEMIP was strongly expressed on the synovial lining (the intima). Moreover, its expression was also present in the subintima. In CPPA biopsies, CEMIP expression was observed in the subintima and in infiltrated inflammatory cells area. Moreover, strong CEMIP expression was also observed in the lining and in blood vessels. In RA biopsies, CEMIP expression was mainly found in the infiltrated inflammatory cell area and blood vessels, the lining being no longer present. αSMA expression was also investigated (Fig. 1b). Mean expression of αSMA was 12.5% in HC, 45.4% (22–73) in OA, 45.1% (23.5–86) in CPPA and 52.2% (26–71.8) in RA synovial membranes. αSMA expression pattern was quite similar to CEMIP expression in the four biopsy types except for the lining of OA synovial membranes where αSMA was less expressed than CEMIP.

CEMIP expression was then investigated in inflamed mouse synovial membrane. To this end, collagenase-induced osteoarthritis (CIOA) mice model was used. Briefly, collagenase was injected twice in the left knee joint. NaCl was injected in their right knee joint and served as control. To analyze inflamed synovial membrane, mice were killed 1 week later (Fig. 1c). Collagenase injection induced thickening of synovial membranes (Fig. 1d) and production of pro-inflammatory cytokines (*Il1b* and *Il6*) (Fig. 1e). mRNA *Cemip* and *Acta2* expressions were also increased in synovial membrane of CIOA knee compared to the paired control knee (Fig. 1e). Mean, standard deviation with range and effect size of these results are presented in Table 1. All effect sizes were between 1.0 and 1.6 (large to huge).

**Table 1:** Synovial thickness and relative gene expression in healthy (NaCl) and inflamed (CIOA) mouse synovial membranes

	Synovial thickness (*v*m)	Relative gene expression			
		*Il1b*	*Il6*	*Acta2*	*Cemip*
NaCl	40 ± 28.58 (18—123)	0.0046 ± 0.0022 (0.0008—0.0071)	0.0008 ± 0.0001 (0.0005–0.0011)	0.0135 ± 0.0067 (0.0089—0.0258)	0.0792 ± 0.0326 (0.0509–0.1284)
CIOA	251 ± 134.5 (60—528)	0.0125 ± 0.0073 (0.0060–0.0225)	0.0094 ± 0.0087 (0.0028–0,0264)	0.0238 ± 0.0100 (0.0144–0.0419)	0.1807 ± 0.0672 (0.0652–0.2572)
*n*	12	6	6	6	6
*κ*	1.4 (0.53–2.32)	1.1 (0.11–2.31)	1.0 (0.20–2.2)	1.6 (0.30–2.90)	1.4 (0.130–2.66)

Values are presented as mean ± standard deviation, with range in brackets. *κ* (Cohen coefficient) with 95% CI in brackets define the effect size as follow: very small (0.01); small (0.2); medium (0.5); large (0.8); very large (1.2) and huge (2)

### In vivo CEMIP silencing decreased synovial OA features induced by collagenase injection

**Fig. 2:**
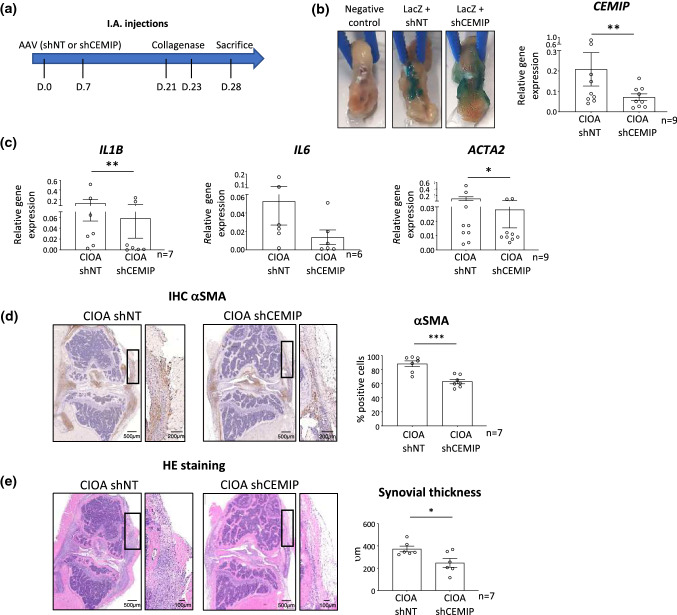
Effect of CEMIP silencing on OA features in a CIOA mouse model. Schematic representation of I.A. injections of AAV and collagenase in mice (**a**). Validation of AAV infection by β-galactositase staining of mouse knee joints infected with AAV vectors carrying LacZ gene and non-target shRNA (LACz + shNT) or CEMIP shRNA (LacZ + shCEMIP). Knee without AAV injection served as negative control (**b**, left). Validation of CEMIP silencing by RT-qPCR analysis of *CEMIP* gene (*n* = 9) in synovial membrane infected with AAV vectors carrying non-target shRNA (shNT) or CEMIP shRNA (shCEMIP) and injected with collagenase (**b**, right). RT-qPCR analysis of *IL1B* (*n* = 7), *IL6* (*n* = 6) and *ACTA2* (*n* = 9) in synovial membrane infected with AAV vectors carrying non-target shRNA (shNT) or CEMIP shRNA (shCEMIP) and injected with collagenase (**c**). Representative histological pictures of mouse synovial membrane sections from CIOA mice having received non-target shRNA (shNT) or CEMIP shRNA (shCEMIP) (*n* = 7). αSMA expression was quantified from sections stained with anti- αSMA antibody and is expressed in percentage of positively stained cells (**d**). Synovial thickness was measured from hematoxylin–eosin coloration and expressed in μm (**e**). Data are expressed as mean with SEM. Parametric paired *t* tests (for values that pass normality test) or non-parametric Wilcoxon tests (for values that did not pass normality test) were applied. *p < 0.05, **p < 0.01 and ***p < 0.001

To study the role of CEMIP in synovial membrane of CIOA mice, adeno-associated virus (AAV) intra-articular (I.A.) injections were performed followed by collagenase I.A. injections (Fig. 2a). In one knee, mice received AAV carrying LacZ expression vector and specific shRNA targeting *Cemip* (sh*Cemip* #1 or #2). In the other knee, mice received AAV containing LacZ expression vector and non-target (NT) shRNA. Collagenase was then injected into both knees. AAV infected tissues were macroscopically visualized by a blue color after β-galactosidase staining (Fig. 2b, left). *Cemip* silencing in synovial membranes was confirmed by RT-qPCR analysis. *Cemip* mRNA level was significantly decreased in synovial membranes infected by AAV-sh*Cemip* compared to synovial membranes infected by AAV-shNT (Fig. 2b, right). Pro-inflammatory cytokines (*Il1b* and *Il6* mRNA) and *Acta2* mRNA expression levels were then investigated in the synovial membrane of CIOA mice where *Cemip* expression was silenced (CIOA-sh *Cemip*) or not (CIOA-shNT). mRNA levels *of Il1b, Il6* and *Acta2* were all decreased in synovial membranes infected by sh*Cemip* compared to shNT (Fig. 2c). αSMA expression was also visualized at protein level. Representative histological pictures and quantification (percentage of positively stained cells) are presented. αSMA expression was significantly decreased in synovial membrane where *Cemip* was silenced (Fig. 2d). Of importance, hyperplasia of synovial membrane induced by collagenase injection was also lower in synovial membrane infected with sh*Cemip* (Fig. 2e). Mean, standard deviation with range and effect size of these results are presented in Table 2. All effect sizes were between 0.5 and 2.7 (medium to huge).

**Table 2:** Relative gene expression, percentage of αSMA positives cells and synovial thickness in inflamed mouse synovial membranes where Cemip expression was silenced (CIOA shCemip) or not (CIOA shNT)

	Relative gene expression				% αSMA positives cells	Synovial thickness (*v*m)
	*Cemip*	*Il1b*	*Il6*	*Acta2*		
CIOA shNT	0.2082 ± 0.2459 (0.0410–0.7680)	0.1246 ± 0.1890 (0.0030–0.5160)	0.0586 ± 0.0684 (0.0020–0.1710)	0.1005 ± 0.1796 (0.0040–0.5070)	88.49 ± 11.05 (70.07–98.01)	372.8 ± 62.33 (322–482)
CIOA shCemip	0.0714 ± 0.0498 (0.0190–0.1640)	0.0581 ± 0.0970 (0.0001–0.2360)	0.0062 ± 0.0081 (0.0010–0.0200)	0.0280 ± 0.0381 (0.0051–0.0100)	63.18 ± 8.430 (52.57–74.59)	248.3 ± 96.96 (115–368)
*n*	9	7	6	9	7	7
*κ*	0.6 (0.34–1.54)	0.4 (0.65–1.45)	0.7 (0.4–1.86)	0.5 (0.43–1.43)	2.7 (1.25–4.14)	0.9 (0,20–2)

Values are presented as mean ± standard deviation, with range in brackets. *κ* (Cohen coefficient) with 95% CI in brackets define the effect size as follow: very small (0.01); small (0.2); medium (0.5); large (0.8); very large (1.2) and huge (2)

### CEMIP depletion deregulated EMT and inflammation response pathways in OA human FLS

**Fig. 3:**
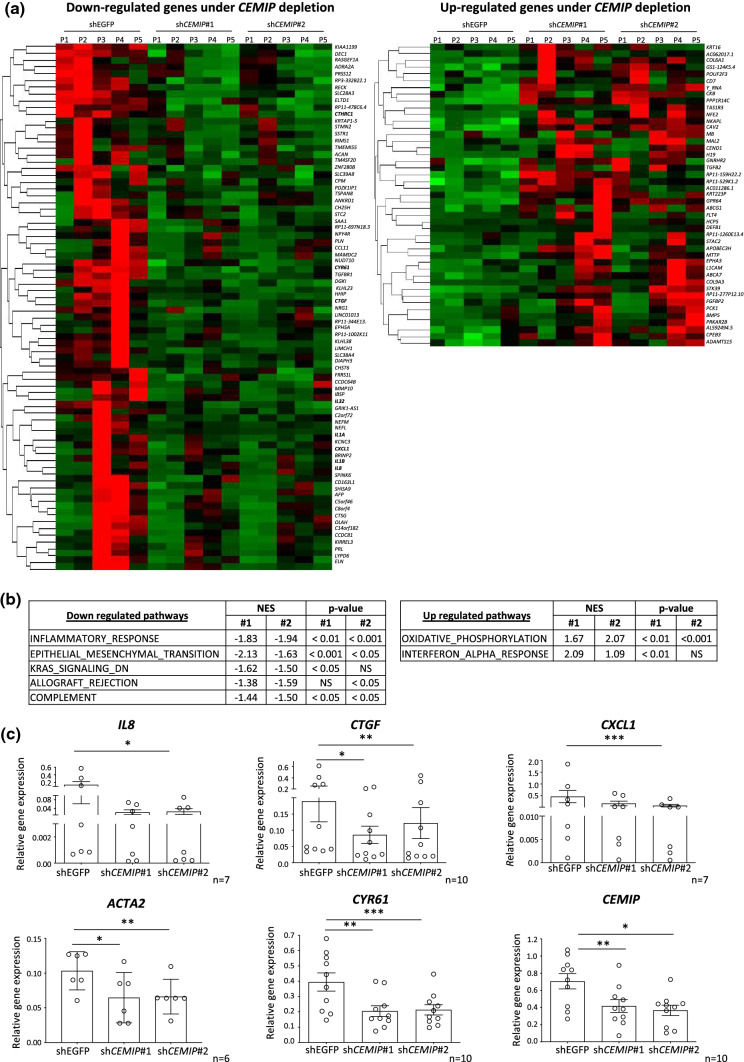
RNA-sequencing and GSEA analysis of Cemip depleted and not depleted human OA FLS. Heatmap of down-regulated (left) and up-regulated (right) genes after CEMIP depletion in OA FLS from 5 patients for genes with a fold change >  ± 2 and a *p* value adjusted < 0.05 (**a**). GSEA analysis of statistically significant pathways down-regulated (left) and up-regulated (righ) by Cemip depletion using Hallmark pathway database (**b**). RT-qPCR analysis of *IL8* (*n* = 7), *CTGF* (*n* = 10), *CXCL1* (*n* = 7), *CYR61* (*n* = 10) and *CEMIP* (*n* = 10) genes in CEMIP-depleted human OA FLS compared to non-depleted cells. Data are expressed as mean with SEM. Parametric paired ANOVA test followed by Tukey post hoc test (for values that pass normality test) or paired Friedman test followed by Dunn’s post hoc test (for values that did not pass normality test) were applied. *p < 0.05, **p < 0.01 and ***p < 0.001

To investigate how CEMIP could regulate synovial OA features, mRNA of human OA FLS was profiled after *CEMIP* depletion (using 2 different specific shRNAs against *CEMIP*: sh*CEMIP* #1 and #2) and compared to control cells (shEGFP). 78 genes were down-regulated and 45 were up-regulated in *CEMIP* depleted cells compared to the control (*p* value adjusted < 0.05 and fold change >  ± 2). Corresponding heatmaps are presented in Fig. 3a. Statistically significant impacted signaling pathways under *CEMIP* depletion were highlighted by Gene Set Enrichment Analysis (GSEA) and are presented in Fig. 3b. The two most up-regulated pathways were the “OXIDATIVE_PHOSPHORYLATION” and the “INTERFERON_ALPHA_RESPONSE” pathways, while the two most down-regulated pathways were the “INFLAMMATORY_RESPONSE” and the “EPITHELIAL_MESENCHYMAL_TRANSITION” (EMT) pathways. Eight genes belonging to these two down-regulated pathways are found in the down-regulated heatmap (in red in Fig. 3a). Graphic representation of corresponding gene counts is presented in Supplementary data 1 *(IL8*, *CTGF*, *CXCL1* (coding for Gro-α), *IL32*, *CYR61* (also called *CCN1*), *CTHRC1*, *IL1A* and *IL1B*). Decreased gene expression of and *CTGF*, *CXCL1* and *CYR61* in Cemip-depleted OA FLS was confirmed by RT-qPCR on another set of patients (Fig. 3c).

### CEMIP induced expression of inflammatory cytokines

**Fig. 4:**
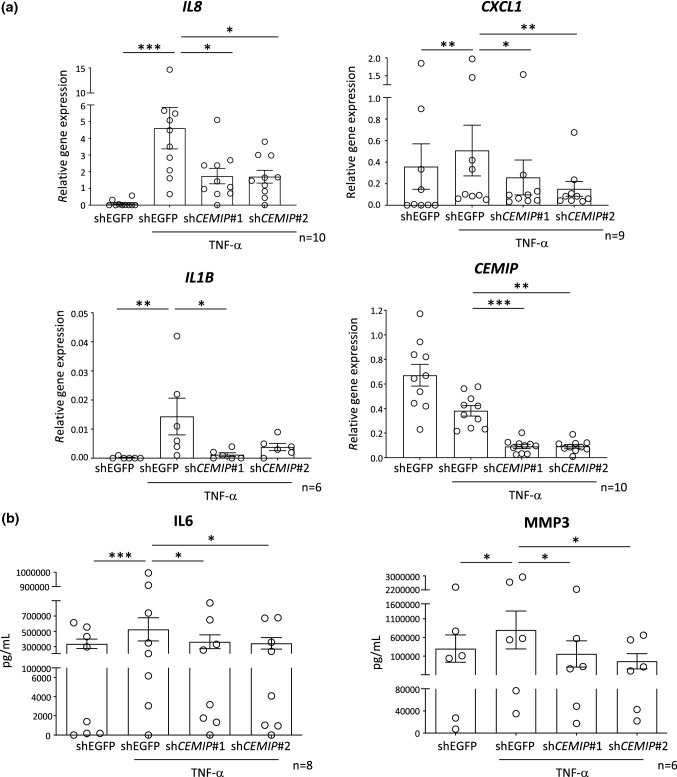
CEMIP regulated pro-inflammatory cytokines in human OA FLS RT-qPCR analysis of *IL8 *(*n* = 10),* CXCL1 *(*n* = 9),* IL1B *(*n* = 6) and *CEMIP *(*n* = 10) genes in CEMIP-silenced FLS (shCEMIP#1 and shCEMIP#2) compared to non-silenced cells (shEGFP) treated or not with TNF-α for one day (**a**). ELISA analysis of IL-6 (*n* = 8) and MMP-3 (*n* = 6) secretion from CEMIP-silenced FLS (shCEMIP#1 and shCEMIP#2) and non-silenced cells (shEGFP) treated or not with TNF-α for one day (**b**). Data are expressed as mean with SEM. Parametric paired ANOVA test followed by Tukey post hoc test (for values that pass normality test) or non-parametric paired Friedman test followed by Dunn’s post hoc test (for values that did not pass normality test) were applied. *p < 0.05, **p < 0.01 and ***p < 0.001

CEMIP expression was observed in inflamed synovial membranes, and in vivo *CEMIP* silencing reduced inflammatory cytokine levels in synovial membranes of CIOA mice. Moreover, RNASeq analysis highlighted that *CEMIP* depletion down-regulated the “INFLAMMATORY_RESPONSE” pathway. Therefore, its role in the regulation of inflammatory cytokines in human OA FLS was investigated. To this end, OA FLS were stimulated with TNFα after *CEMIP* depletion, and mRNA levels expression of *IL8, CXCL1* and *IL1b* was analyzed. *All* mRNA levels were up-regulated by TNF-α stimulation in control cells (shEGFP). When *CEMIP* was silenced, mRNA level of all genes was decreased (Fig. 4a). Of note, *CYR61* mRNA level was not modulated by TNF-α stimulation (data not shown). Moreover, interleukin-6 (IL-6) and MMP-3 secretion was monitored on cell supernatants treated with sh*CEMIP* (#1 and #2) or shEGFP (control cells) and stimulated or not with TNF-α for 1 day. Both IL-6 and MMP-3 secretions were increased under TNF-α stimulation in cells expressing *CEMIP* (control cells). However, this secretion was significantly decreased in cells where *CEMIP* expression was silenced (Fig. 4b). Of note, secretions of IL-6 and MMP-3 were increased after 7 days of TGF-β stimulation and decreased in *CEMIP*-silenced fibroblasts compared to control cells (Supplementary data 2).

### CEMIP induced FLS proliferation and fibrosis through the TGF-β pathway

**Fig. 5:**
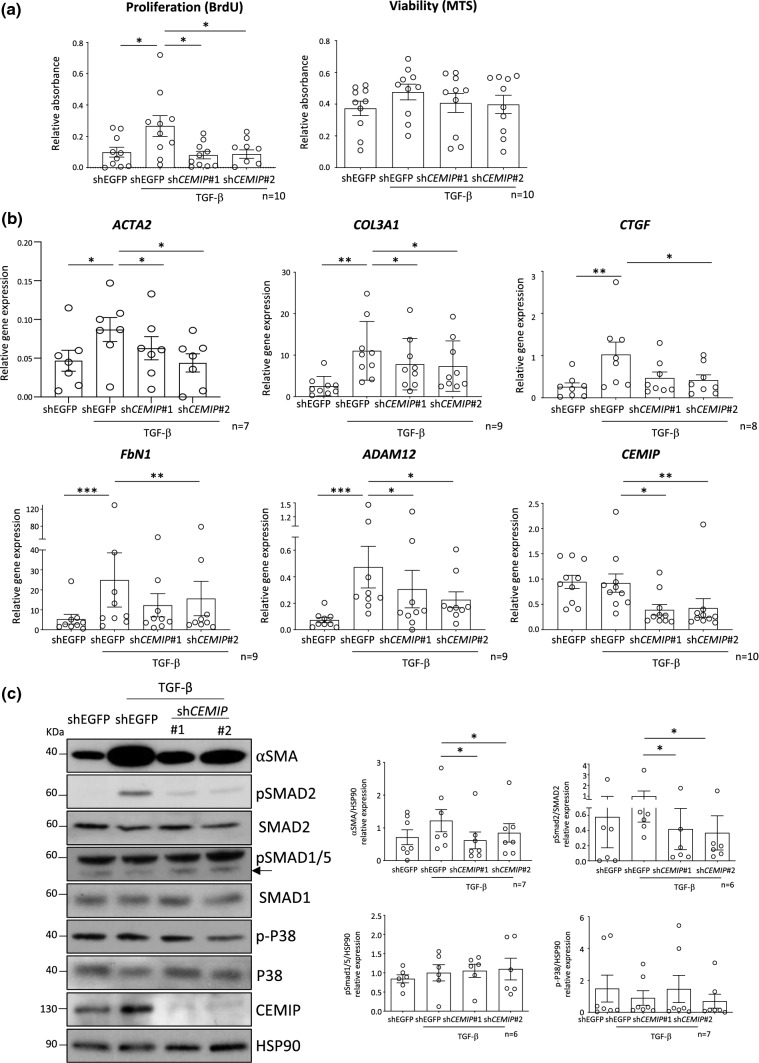
CEMIP regulates FLS proliferation, fibrosis markers and TGF-β signaling. Proliferation (BrdU) (*n* = 10) and viability (MTS) (*n* = 10) tests performed on CEMIP-silenced FLS (shCEMIP#1 and shCEMIP#2) compared to non-silenced cells (shEGFP) treated or not with TGF-β for 2 days (**a**). RT-qPCR analysis of *ACTA2 *(*n* = 7), *COL3A1 *(*n* = 9),* CTGF *(*n* = 8), *FbN1 *(*n* = 9), *ADAM12 *(*n* = 9)* and CEMIP* (*n* = 10) genes in CEMIP-silenced FLS (shCEMIP#1 and shCEMIP#2) compared to non- silenced cells (shEGFP) treated or not with TGF-β for 7 days (**b**). Western blot analysis of αSMA (*n* = 7), pSmad2 (*n* = 6), pSmad1/5 (*n* = 7), p-p38 (*n* = 7) and CEMIP (*n* = 7) in CEMIP-silenced cells (shCEMIP#1 and shCEMIP#2) compared to non-silenced cells (shEGFP) treated or not with TGF-β for 7 days. Western blot quantifications illustrate the expression of the different proteins normalized to non-phosphorylated form of proteins or HSP90 expression (loading control) (**c**). Data are expressed as mean with SEM. Parametric paired ANOVA test followed by Tukey post hoc test (for values that pass normality test) or non-parametric paired Friedman test followed by Dunn’s post hoc test comparisons (for values that did not pass normality test) were applied. *p < 0.05, **p < 0.01 and ***p < 0.001

Hyperplasia is a hallmark of osteoarthritis. Here, we showed in vivo *Cemip* depletion decreased synovial hyperplasia of CIOA mice. In vitro *CEMIP* depletion in FLS also reduced cell proliferation induced by TGF-β stimulation. Of note, *CEMIP* silencing did not affect cell viability (Fig. 5a).

In addition to inflammation and hyperplasia, OA synovial membrane is also characterized by fibrosis. Previously, we showed that CEMIP regulates fibrosis in OA cartilage [16]. In this work, we observed that CEMIP is expressed along with αSMA in human inflamed synovial membranes, and in vivo *Cemip* silencing induced a decrease of αSMA expression in synovial membranes of CIOA mice. Finally, GSEA analysis underscored that *CEMIP* depletion deregulated the EMT pathway. Of note, FLS exhibiting a stem cell-like phenotype would rather be an EMT-like phenomenon. The role of CEMIP was therefore studied in synovial fibrosis. To this end, mRNA level expression of genes implicated in fibrosis were investigated in *CEMIP* silenced human OA FLS (sh*CEMIP*#1 and #2) compared to control cells (shEGFP) stimulated with TGF-β for 7 days. *ACTA2, COL3A1, CTGF, FbN1* and *ADAM12* relative mRNA expression were all increased by TGF-β stimulation in cells expressing *CEMIP* (control cells). However, *CEMIP* silencing induced a decrease in the expression of these five genes (Fig. 5b). At protein level, αSMA expression induced by TGF-β stimulation was decreased in cells where *CEMIP* expression was abolished (Fig. 5c). After 1 day of TGF-β stimulation, *ACTA2* relative mRNA expression was decreased in Cemip-silenced cells, but not *COL3A1*, *CTGF* and *FbN1* relative mRNA expression. At protein level, αSMA and pSmad2 expression was also decreased in Cemip-silenced cells, but not pP38 and pSmad1/5 (Supplementary data 3).

The most well-known fibrosis-inducing signaling pathway is the TGF-β pathway. The role of CEMIP in the regulation of this pathway was therefore investigated. To this end, phosphorylation of SMADs (canonical pathway) and P38 (non-canonical pathway) was analyzed. After 7 days of TGF-β stimulation, pSMAD2 expression was increased while pSMAD1/5 and p-P38 expression remained stable. The silencing of *CEMIP* induced a decrease in the expression of pSMAD2 and had no effect on pSMAD 1/5 and p-P38 (Fig. 5c).

### Regulation of CEMIP expression

**Fig. 6:**
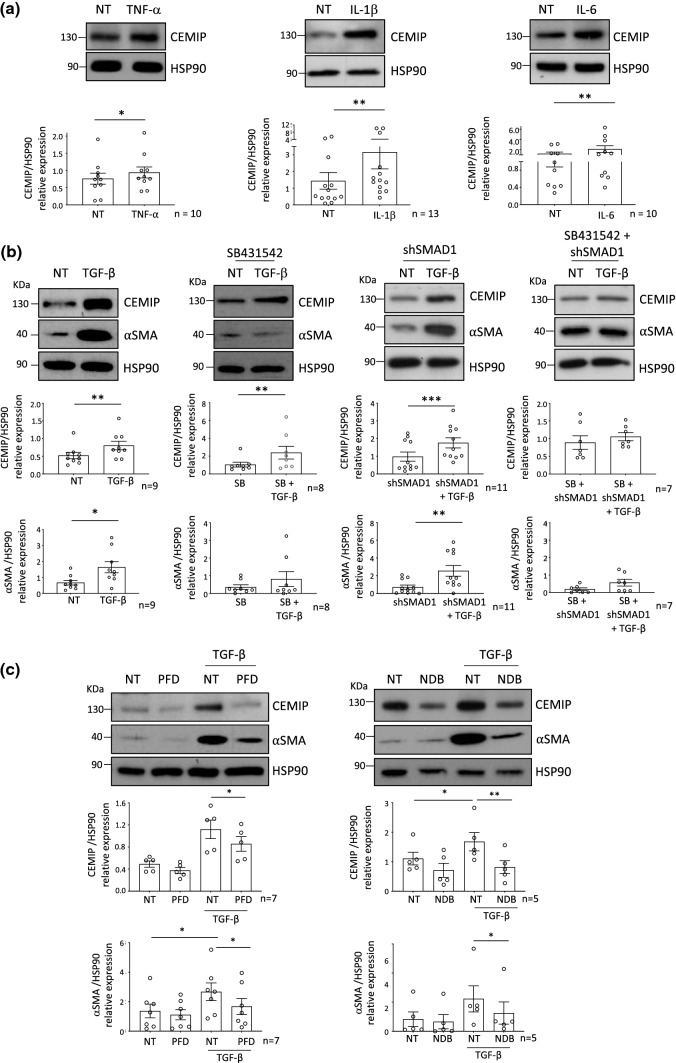
Regulation of CEMIP expression. Western blot analysis of CEMIP expression after 1 day of TNF-α (*n* = 10), IL-1β (*n* = 13) and IL-6 (*n* = 10) stimulation (**a**). Western blot analysis of CEMIP and αSMA expression after 7 days of TGF-β stimulation (*n* = 9); after treatment with SB431542 (SB) and with or without TGF-β for 7 days (*n* = 8); in SMAD1 silenced cells (shSMAD1) treated or not with TGF-β for 7 days (*n* = 11) and in SMAD1 silenced cells (shSMAD1) pretreated with SB431542 (SB) and treated or not with TGF-β for 7 days (*n* = 7) (**b**). Western blot analysis of CEMIP and αSMA expression after Pirfenidone (PFD) treatment with or without TGF-β stimulation (*n* = 5) and after Nintedanib (NDB) treatment with or without TGF-β stimulation (*n* = 5) (**c**). Western blot quantifications illustrate the expression of the different proteins normalized to HSP90 expression (loading control). Data are expressed as mean with SEM. Parametric paired *t* test, for two groups comparisons or paired ANOVA test followed by Tukey post hoc test, for multiple comparisons (for values that pass normality test) or non-parametric Wilcoxon test, for two group comparisons or paired Friedman test followed by Dunn’s post hoc test, for multiple comparisons (for values that did not pass normality test) were applied. *p < 0.05, **p < 0.01 and ***p < 0.001

Modulation of CEMIP expression by pro-inflammatory cytokines and pro-fibrosis pathway (TGF-β pathway) was then investigated. To this end, OA FLS were stimulated with TNF-α, IL-1β and IL-6. All these stimulations increased CEMIP expression (Fig. 6a).

To know if TGF-β pathway could modulate CEMIP expression, cells were stimulated with TGF-β for 7 days. CEMIP expression was increased in the same way as αSMA in TGF-β stimulated cells compared to non-treated (NT) cells (Fig. 6b). Of note, longer stimulation, up to 21 days, did not increase CEMIP expression compared to 7 days (Supplementary data 4a). Cells were then pre-treated with specific inhibitors followed by TGF-β stimulation or not. SB431542 was used to inhibit the pSMAD2/3-Alk5 pathway of TGF-β signaling. When the pSMAD2/3-Alk5 pathway was inhibited, TGF-β stimulation increased CEMIP expression, but did not modify αSMA expression (Fig. 6b). To inhibit the p-pSMAD1/5-Alk3 pathway of TGF-β signaling, SMAD1 was silenced using specific shRNA (Supplementary data 4b). Cells were then stimulated or not with TGF-β stimulation. In these conditions, TGF-β stimulation induced both CEMIP and αSMA expression (Fig. 6b). Non-canonical TGF-β signaling was investigated by concomitant inhibition of both TGF-β canonical pathways (pSMAD2/3-Alk5 and pSMAD1/5-Alk3 pathway) followed or not by TGF-β stimulation. Both CEMIP and αSMA expressions did not increase after TGF-β stimulation (Fig. 6b). All these data suggest that total inhibition of TGF-β canonical signaling pathway prevents CEMIP expression induced by TGF-β stimulation. Further, TGF-β increased CEMIP expression regardless of whether SMAD pathway was activated (pSMAD2/3-Alk5 or pSMAD1/5-Alk3) in the canonical signaling. Of note, as shown in Fig. 5c, only the pSMAD2/3-Alk5 pathway was activated after 7 days of TGF-β stimulation suggesting that CEMIP expression is preferentially induced by this pathway in our model. In addition, the pSMAD2/3-Alk5 pathway was required to induce αSMA expression.

As CEMIP silencing reduced fibrotic markers in vitro and in vivo, we assessed the effect of two anti-fibrotic drugs on CEMIP and αSMA expression. To this end, cells were pre-treated with pirfenidone (PFD) or nintedanib (NDB) for 1 day followed by TGF-β stimulation for 7 days. CEMIP and αSMA expressions up-regulated by TGF-β stimulation were decreased by both pirfenidone and nintedanib treatment (Fig. 6c).

## Discussion

OA is the most common chronic joint disease in adults and is characterized by joint pain, short-lived morning stiffness and functional limitations [25]. The main physiological features are cartilage degradation, synovial inflammation, subchondral bone erosion and osteophyte formation. OA was for long considered as a purely degenerative disease. However, it is increasingly recognized that synovitis plays an initial and important role in OA development [26]. Indeed, synovial inflammation appears in the early stages of the disease and persists to the late stages [27]. It is activated by cartilage degradation that releases pro-inflammatory mediators into the synovial cavity [3]. In return, synoviocytes produce pro-inflammatory molecules that attract immune cells, induce angiogenesis and sustain cartilage degradation [3]. Moreover, OA synovitis shares similar features with RA synovitis, i.e., hyperplasia, mononuclear cells infiltration, neo-angiogenesis and fibrosis [5, 27]. Fibrosis is observed at the late stages of the disease and results from chronic inflammation and tissue injury [5, 7, 28]. Synovial fibrosis is characterized by the transformation of fibroblasts into myofibroblasts that proliferate intensively and produce excessive collagen and αSMA [5, 8]. As a consequence, the synovial membrane becomes hyperplasic and rigid contributing to joint pain and functional limitations. Therefore, targeting synovitis and particularly hyperplasia, inflammation and fibrosis appears essential to slow down disease progression.

In this work, we showed that CEMIP expression was up-regulated in inflamed synovial membranes. CEMIP was overexpressed in human synovial membrane from OA, CPPA and RA patients compared to healthy synovial membrane. CEMIP was principally expressed in blood vessels of healthy synovium. In the three types of inflamed synovium, CEMIP showed different expression pattern. In OA and CPPA, CEMIP was intensely expressed in the lining. Moreover, CEMIP was also observed in the subintima and co-localized with αSMA, a fibrosis marker. In RA synovial membranes, CEMIP was principally expressed in the subintima and in immune cell infiltrates, the lining being dismantled. Moreover, *Cemip* was also overexpressed in inflamed synovial membranes of CIOA mice compared to control mice, as also observed for αSMA and pro-inflammatory cytokines. High CEMIP expression has been described in synovial membrane from RA patients and more recently from OA patients [18, 19, 22]. Recently, we and others have shown that CEMIP is also overexpressed in cartilage of OA patients [16, 20, 21, 24]. Moreover, we showed that CEMIP induces a fibrosis-like process in OA chondrocytes [16].

The role of CEMIP in the induction of synovitis was therefore explored in vivo. *Cemip* silencing in knee of CIOA mice decreased the main features of OA synovial membrane. First, inflammation, monitored by *Il1b* and *Il6* expression was lower in synovial membrane of CIOA mice when *Cemip* expression was silenced. Second, fibrosis, evaluated with αSMA (or *Acta2* mRNA) expression was also decreased in synovial membrane of CIOA mice when *Cemip* expression was silenced. Finally, we demonstrated that *Cemip* silencing reduced synovial membrane hyperplasia induced by collagenase injection.

To go further in deciphering the role of CEMIP, high-throughput RNA-sequencing analysis followed by GSEA was performed on human OA FLS depleted or not for *CEMIP*. First, we observed that *CEMIP* depletion down-regulated the inflammatory response pathway and pro-inflammatory cytokine expression. Moreover, TNF-α—induced expressions of *IL-6*, *IL-8*, *MMP-3* and *CXCL1* were decreased in CEMIP-silenced cells. In human OA chondrocytes, CEMIP was previously shown to induce the expression of inflammatory cytokines (IL-6, TNF-α and PGE2) [20, 24]. CEMIP also mediated IL-1β expression through Erk pathway activation and NF-KB translocation in chondrosarcoma cell line [21]. This induction of inflammation could be explained by the role of CEMIP in HA depolymerization. Indeed, CEMIP induced degradation of high HA in skin and arthritis synovial fibroblasts through its N-terminal sequence, leading to small HA fragments enhancing inflammation [18, 29, 30]. Second, GSEA analysis also underscored that *CEMIP* depletion down-regulated the EMT pathway. In this context, TGF-β induced the expressions of *ACTA2, COL3A1, CTGF, FbN1 and ADAM12* mRNA as well as of αSMA protein that were decreased after *CEMIP* silencing. Moreover, CEMIP regulated the pro-fibrotic side of the TGF-β signaling. Indeed, SMAD2 phosphorylation induced by TGF-β stimulation was decreased when *CEMIP* was silenced, whereas pSMAD1/5 and p-P38 were not modulated by CEMIP expression. Recently, we highlighted the potential role of CEMIP in fibrosis induction of OA chondrocytes [16]. The role of CEMIP in epithelial-to-mesenchymal transition of cancer cells is already well known [31, 32]. Moreover, CEMIP induces fibrosis of Crohn disease fibroblasts through the depolymerization of HA into small HA fragments with pro-inflammatory and pro-fibrotic properties [23]. Recently, high plasma level of CEMIP was found in patients suffering from idiopathic pulmonary fibrosis (IPF) compared to controls. Interestingly, this level decreased after 7 months of pirfenidone treatment, an anti-fibrotic drug [33].

It is worth noting that stimulation with TGF-β induces FLS proliferation in the presence of *CEMIP,* whereas it was abrogated when *CEMIP* was silenced. It could be explained by the role of CEMIP in synovial membrane hyperplasia observed in our in vivo model.

All these data suggest that CEMIP induced inflammation, hyperplasia and fibrosis, the three main characteristics of OA synovial membrane. Therefore, targeting CEMIP expression could slow down OA progression.

Regulation of CEMIP seems to be cell-type specific. Indeed, the increase of CEMIP expression is observed after stimulation with TNF-α in OA chondrocytes, IL-6 in Crohn’s disease fibroblasts and IL-1β in pancreatic ductal adenocarcinoma cell line [20, 23, 34]. However, IL-1β and TNF-α have no effect on CEMIP in skin fibroblasts and Crohn’s disease fibroblasts [18, 23]. Short stimulation with TGF-β on skin fibroblasts and OA chondrocytes decreases the expression of CEMIP protein, while no effect is observed on OA chondrocytes at mRNA level [16, 18, 20]. In OA synoviocytes, we observed that IL-1β, TNF-α and IL-6 all increased CEMIP expression. Moreover, long-time stimulation with TGF-β also increased CEMIP expression through the TGF-β /p-Smad2/3-Alk5 pathway. Therefore, we tested anti-fibrotic drugs on CEMIP expression and observed that both pirfenidone and nintedanib decreased CEMIP expression after TGF-β stimulation. Very recently, Wei et al. showed that pirfenidone reduces inflammation and fibrosis in rabbit OA synovial membrane [35].

Both inflammation and fibrosis induce joint stiffness and pain, making them essential therapeutic targets. Here, we showed that Cemip induces both inflammation and fibrosis of synovial membrane. This suggests that Cemip could have an essential role in the early stage of OA. These data strengthen our previous study, showing that Cemip expression was responsible for OA cartilage fibrosis induction [16] and reveal a key role for Cemip in different OA joint tissues. Therefore, targeting Cemip would allow to manage OA disease at an early stage and would contribute to reduce joint stiffness and pain. Moreover, our results showed that the use of anti-fibrotic drugs like pirfenidone and nintedanib could achieve this purpose.

Other diseases share similar mechanisms, for which inflammation and fibrosis play an important role (i.e., systemic sclerosis, idiopathic pulmonary fibrosis or Crohn disease). Therefore, beyond osteoarthritis, it would be relevant to assess the potential role of Cemip in these different pathologies.

### Limitations

There are some limitations in this study that should be considered. The expression of Cemip in synovial membranes could only be measured for one healthy control. Therefore, we cannot draw statistically relevant conclusions. However, the use of a mouse model allows to partially overcome this issue. Nevertheless, the CIOA mouse model is an inflammatory OA model that does not represent the heterogeneity of all human OA phenotypes (biomechanics, osteoporotic, metabolic and inflammatory). Moreover, AAV infection may infect other tissues of the joint in addition to the synovial membrane (muscle, tendon, fat), which could therefore induce an indirect effect on the physiopathology of the membrane. This in vivo model is based on the decrease of CEMIP expression. The overexpression model could also be interesting to investigate in order to decipher the role of CEMIP in the different stages of OA development. Finally, TNF-Α was used to induce inflammation on FLS. However, IL1-β and IL-6 could have been used as pro-inflammatory mediators in OA development, being also involved in Cemip expression.

## Methods

### Subject recruitment

Synovial membrane biopsies used for immunochemistry were obtained from the biobank (CHU Sart-Tilman, ULiège). There was one healthy control (man, 81 years old, BMI: 25.43 kg/m^2^), nine OA patients (89% of women, mean age 57.4, range 36–89) years and mean BMI 30.3 (18–42 kg/m^2^), seven CPPA patients (71% of women, mean age 64.6, range 50–74) years and mean BMI 24.2 (22–33.8 kg/m^2^) and eight RA patients (63% of women, mean age 55, range 29–78) years and mean BMI 26.5, range 16.4–33.9) kg/m^2^). Age and sex were not statistically different between OA, CPPA and RA patients. Synovial inflammation scoring was based on Tak’s score by the sum of the following components: synovial hyperplasia (hy; 0–4 score), infiltration degree of lymphocytes (ly; 0–4 score), plasma cells (pl; 0–4 score), polymorphonuclear cells (PMN; 0–3 score) and macrophages (with CD68 expression, 0–3 score) (Table 3).

**Table 3:** Demographic and clinical characteristics of patients used for synovial biopsies analysis

	HC	OA	CPPA	RA
*n*	1	9	7	6
Age [mean (interval)]	81	57.4 (36–89)	64.6 (50–74)	57.6 (45–78)
% of woman	0% (0/1)	89% (8/9)	71% (5/7)	67% (4/6)
BMI [mean (interval)]	25.43	30.3 (18–42)	24.2 (22–33.8)	24.1 (16.4–33.9)
K&L score [median (interval)]		3 (0–4)	2 (0–4)	
Histological inflammatory score		4 (3–8)	5 (5–13)	14.5 (12–17)

Synovial membranes used for FLS isolation were obtained in collaboration with the Orthopedic Surgery department (CHU Sart-Tilman, ULiège) (*n* = 80; 53% females and 47% males) undergoing knee replacement surgery. Mean age was 65.5 (49–83) years and mean BMI was 29.2 (18–42.1) kg/m^2^.

### Animal experimentation procedures

8 week-old C57BL6 mice were used for the study. As it is well-known that OA can be induced to a greater extent in males than in females in OA mice models, only males were used in this study [36, 37]. Mice were anesthetized using isoflurane and knees were sanitized prior injections. For CIOA generation, collagenase VII (1U per articulation, Sigma-Aldrich, St Louis, Michigan, USA) or NaCl (control mice) was injected using a 30 gauge needle in intra-articular knee cavity two times, 2 days apart. Mice were killed 1 week after the injection. Synovial hyperplasia was measured from hematoxylin–eosin slide by the measurement of synovial thickness using QPath software [38]. The measurement was made at three different locations of the synovium and the average was plotted (Fig. 1D). The mRNA expression of inflammatory cytokines (IL1B and IL6) was also analyzed (Fig. 1E).

### AAV production and injection

AAV plasmids allowing dual expression of LacZ, under the control of cytomegalovirus (CMV) promoter and coding for β-galactosidase, two different shRNA directed against mouse *Cemip* (U6 promoter), as well as a non-target shRNA as control, were purchased at Vector Builder: pAAV CMV LacZ U6 shRNA m*Cemip*#1 (AAV-LacZ-sh*Cemip*#1) (VB190725-1198thb): target sequence AGGATGTTGTGGGCTATAATT, pAAV CMV LacZ U6 shRNA m*Cemip*#2 (AAV-LacZ-sh*Cemip*#2) (VB190725-1201dvw): target sequence CATGCAGGAGGGAGGATATTT pAAV U6 shRNA NT-CMV LacZ (AAV-LacZ-shNT) (VB180803-1034gja): target sequence CCTAAGGTTAAGTCGCCCTCG. GIGA Viral vectors platform (ULiège, Liège, Belgium) generated AAV 2/1 vectors (serotype 1) by co-transfecting 293 AAV Cell Line (Cell Biolabs # AAV-100) with these plasmids, AAV-1 Rep-Cap plasmid (Cell Biolabs # VPK-421) and pHelper plasmid (Cell Biolabs, Part No. 340202of VPK-401 kit). AAV were collected from cells and supernatant and then filtered through 0.1 µM filters and finally concentrated using 100 kDa AMICON tubes. AAV were then titrated using qPCR AAV Titration (Titer) Kit (ABM^®^ Good #G931, Rhichmond, BC, Canada) and tested on HEK cell to confirm β-galactosidase expression. Intra-articular injection of 5E + 11 TU of each AAV type was performed twice a week apart in the knee of mice. AAV-LacZ-shNT was injected in the right knee, while AAV-LacZ-sh*Cemip*#1 or #2 was injected in the left knee. Two weeks after the second injection, collagenase was injected in each knee. Mice were killed 1 week later.

### β-Galactosidase staining

β-Galactosidase coloration was performed following the Kyostio-Moore et al. protocol [39]. Mouse whole joints were fixed in PFA (2% diluted in 0.1 M NaHPO_4_ pH 7.4) for 1 h and then incubated with X-gal solution (1.2 mg/ml, Invitrogen) overnight at 37 °C. After washing in PBS, samples were fixed in 10% normal buffered formalin (NBF) overnight.

### Immunohistochemistry analysis

Immunohistochemistry analysis was performed on synovial membranes obtained from human and mice. For human samples, tissues were fixed in 4% paraformaldehyde for 24 h, dipped in 70% (v/v) ethanol and embedded in paraffin. For mouse samples, whole knee joints were fixed in 4% paraformaldehyde for 24 h, decalcified in EDTA for 15 days, dipped in 70% (v/v) ethanol and embedded in paraffin. Immunohistochemistry was performed on slide after heating, dewaxing and antigen retrieval with a steamer for 10 min in target retrieval solution (Agilent, Santa Clara, California, USA). Endogenous peroxidases were blocked by incubation with hydrogen peroxide followed by incubation with Dako-Real antibody diluent (Agilent). Sections were incubated (or not for negative controls) with a primary antibody against CEMIP (Santa Cruz, Dallas, Texas, USA) or against αSMA (Agilent for human tissue and from Abcam, Cambridge, Massachusetts, USA for mouse tissues). Sections were then incubated with EnVision + System-HRP labeled polymer (Agilent). Peroxidase was detected with Liquid DAB + Substrate Chromogen System (Agilent) and sections were counterstained with Carazzi’s Hematoxylin (EMD Millipore, Billerica, Massachusetts, USA). Staining was revealed with Nanozoomer Digital Pathology 2.0 HT scanner (Hamamatsu photonics, Hamamatsu, Japan) and quantified using QPath software [38]. Hematoxylin–eosin staining was performed according to classical protocols.

### Cell culture and reagents

Human OA FLS were isolated from knee joints as previously described [40]. Cells were cultured in DMEM medium (with 10% fetal bovine serum, 1% L-glutamine (200 mM), 100 units/ml penicillin and 100 μg/ml streptomycin (BioWhittaker, Walkersville, Maryland, USA)) allowing the selection of FLS and maintained at 37 °C in a 5% CO_2_ atmosphere. Cells were used between passage 3 and 7. FLS were treated with TNF-α (10 ng/mL, Peprotech, London, UK), TGF-β1 (10 ng/ml, GIBCO-BRL, San Francisco, California, USA), SB431542 (1.5 μM, Sigma-Aldrich), pirfenidone (PFD, 1 mM, Sigma-Aldrich) or nintedanib (NDB, 1 μM, Sigma-Aldrich). DMSO was used as vehicle for PFD and NDB stimulations in the same quantities.

### Lentiviral vectors generation and transduction

shRNA plasmids against h*CEMIP*, h*SMAD1* and EGFP were purchased from VectorBuilder (Neu-Isenburg, Germany). Lentiviral vectors were generated by our GIGA Viral Vectors platform as previously described [16]. Briefly Lenti-X 293 T cells (Clontech, Mountain View, California, USA) were co-transfected with a pSPAX2 (Addgene^®^, Cambridge, MA, USA) and a VSV-G encoding vector. Viral supernatants were collected 48 h, 72 h and 96 h post-transfection, filtered (0.2 µM) and concentrated 1000 × by ultracentrifugation. The lentiviral vectors were then titrated with qPCR Lentivirus Titration (Titer) Kit (ABM^®^, LV900).

### RT-qPCR analysis

For in vitro experiments, total RNA extractions were performed using Nucleospin RNA kit (Macherey–Nagel, Düren, Germany). For in vivo experiments, total RNA extractions were done using standard protocol of TRIzol extraction. RNA was reverse transcribed with RevertAid H Minus First Strand cDNA Synthesis Kit (Thermo Scientific, Pittsburgh, Pennsylvania, USA). cDNA was amplified by real-time PCR using the KAPA SYBR FAST detection system (Sopachem, Eke, Belgium) and run on LightCycler 480 instrument (Roche Diagnostics, Mannheim, Germany). Individual real-time PCR efficiencies *E* = 10^(−1/slope)^ were calculated for each primer by the generation of cDNA dilution curves. The 2^−ΔCT^ method was used to calculate the relative gene expression between non-treated (calibrator sample) and treated conditions. Normalization was made with the *GAPDH* endogenous control gene for in vitro experiments and with *Gapdh*, *B2M*, Hsp90, *Hprt* and *Actb* endogenous control genes for in vivo experiments. Primers were purchased from Eurogentec (Seraing, Belgium) or Integrated DNA Technologies (Coralville, Iowa, USA).

### High-throughput RNA-sequencing and gene set enrichment analysis (GSEA)

Libraries were prepared, sequenced and analyzed as previously described [16]. Normalization of RNASeq gene counts and differential expression analyses were performed with DESeq2 (R package DESeq2_1.26.0). Normalized gene counts were used for the pairwise log2Ratios and FoldChanges computing in both shEGFP and sh*CEMIP*#1; and shEGFP and sh*CEMIP*#2 conditions. Genes were filtered based on the Log2Ratios signs and genes with positive Log2Ratios or with negative Log2Ratios in both conditions were kept for downstream analyses. Heatmaps were processed using R software (v3.6). Genes were filtered based on the multivariate test (padj ≤ 0.05), as well as pairwise tests (padj_sh*CEMIP*#1 ≤ 0.05 and padj_sh*CEMIP*#2 ≤ 0.05) using their respective contrasts. Gene values were centered, scaled, and clustered with the Euclidean distance and the complete linkage. Pre-ranked GSEA was performed against the “h.all.v6.2.symbols” gene set database, using the “gsea-3.0 software”.

### Western blot analysis

FLS were lysed in RIPA buffer. After separation by SDS-PAGE, proteins were transferred to polyvinylidene difluoride membranes (EMD Millipore). Membranes were blocked and incubated with anti-CEMIP (Phoenix Pharmaceutical, Burlingame, California, USA), anti-β-CATENIN and HSP90 (Santa-Cruz Technologies) and anti- αSMA, SMAD1, p-SMAD2, pSMAD1/5 and p-P38 (Cell Signaling, Beverly, Massachusetts, USA). Membranes were then incubated with anti-mouse or anti-rabbit secondary antibodies conjugated with peroxidase (Cell Signaling). Signal was revealed using the enhanced chemiluminescence detection reagent (ECL kit, Thermo Fisher Scientific, Waltham, Massachusetts USA) and analyzed by densitometry. The intensity of each band was assessed with Image Studio Lite Software (Li-Cor Biosciences, Linkolin, Nebraska, USA) and normalized with the intensity of the corresponding anti-HSP90 signal used as an internal standard.

### ELISA

Commercially available sandwich enzyme-linked immunosorbent assays were used for IL-6 and MMP-3 quantification in the cell supernatant according to manufacturer’s instructions (R&D Systems, Minneapolis, Minnesota, USA). The calibration ranges were 9.38–600 pg/mL and 31.3–2000 pg/mL for IL-6 and MMP-3, respectively. All measurements were performed in triplicate.

### Viability and proliferation assays

FLS were treated with anti-CEMIP shRNA or control shRNA and stimulated or not with TGF-β for 2 days. Cell viability was assessed by using MTS assay (Promega, Madison, Wisconsin, USA) and cell proliferation was measured by using BrdU assay (Abcam) according to the manufacturer’s instructions. Results are expressed as a percentage of surviving (for MTS test) or proliferating (for BrdU test) cells compared with control cells.

### Statistical analysis

Kolmogorov–Smirnov test was used to assess values distribution. For values that pass normality test, parametric tests were applied: paired *t* test for two group comparisons and paired ANOVA test followed by Tukey post hoc test for multiple comparisons. For values that did not pass normality test, paired non-parametric tests were applied: paired Wilcoxon test for two group comparison and paired Friedman test followed by Dunn’s post hoc test for multiple comparisons. Results were considered significant at the 5% critical level (* = *p* < 0.05, ** = *p* < 0.01, *** = *p* < 0.001). Calculations and graphs (mean with SD) were generated with GraphPad Prism software (version 5.0, La Jolla, California, USA).

### Study approval

Human synovial membrane collection used for immunohistochemistry or FLS extraction was approved by the ethical committee (B707201732662; ref: 2017/147) and the biobank research committee (BB190058) of CHU of Liège. All animal experimentation procedures were approved by the local ethical committee (LA1610002; #14-1721 University of Liège, Liège, Belgium).

## Supplementary Information

Below is the link to the electronic supplementary material.

Supplementary file1 (PDF 247 KB)

## Data Availability

The datasets generated during and/or analyzed during the current study are available from the corresponding author on reasonable request.
