# Supplementary material for: CEMIP (KIAA1199) regulates inflammation, hyperplasia and fibrosis in osteoarthritis synovial membrane
Source: Cell Mol Life Sci. 2022 Apr 27;79(5):260. doi: 10.1007/s00018-022-04282-6 (PMC9042994; doi:10.1007/s00018-022-04282-6)
Supplement: Supplementary file 1 — Supplementary file1 (PDF 247 KB) [file 18_2022_4282_MOESM1_ESM.pdf]

Supplementary data 1

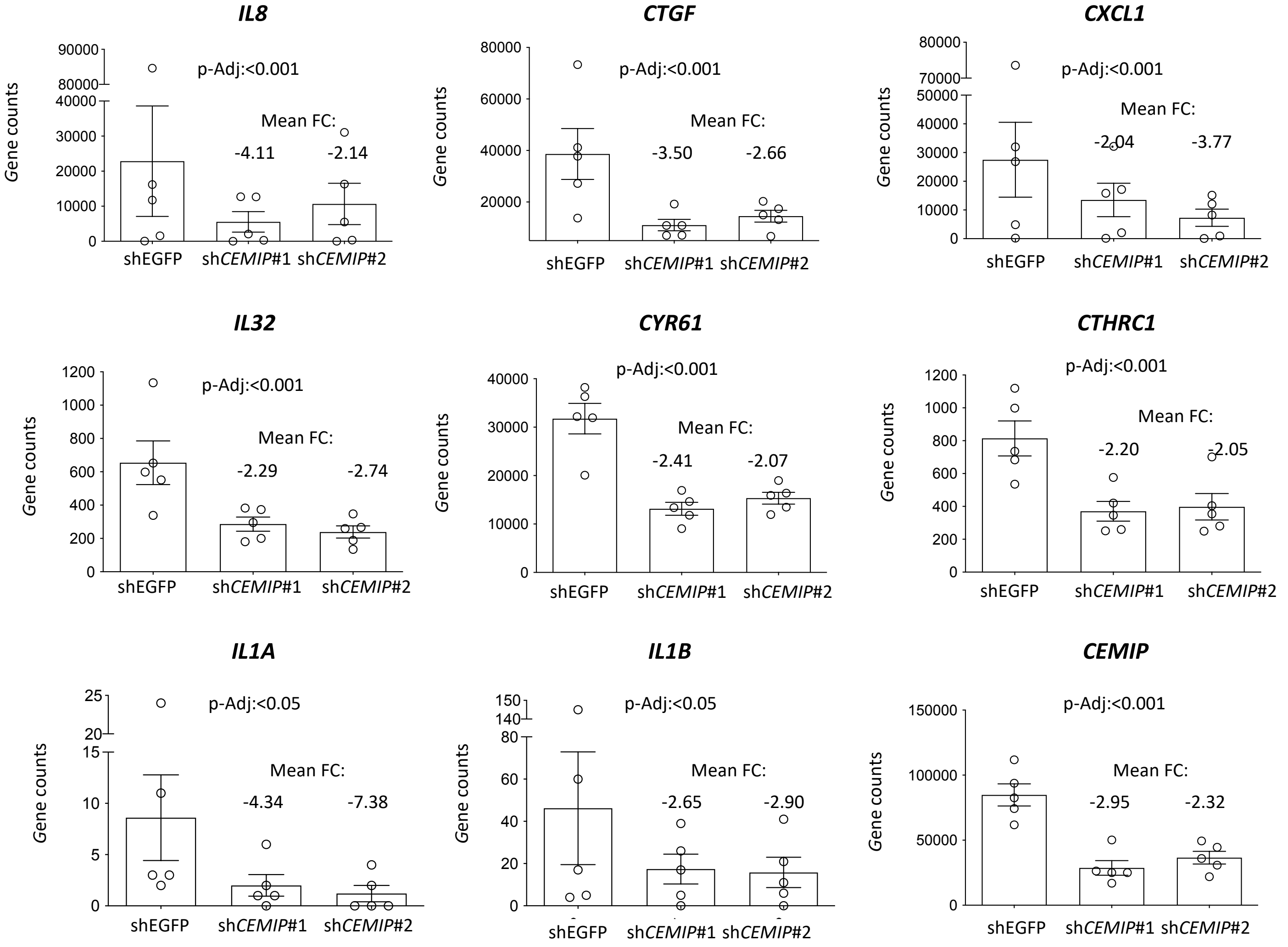

CEMIP depletion decreased gene expressions belonging to inflammatory response and EMT pathways

Normalized gene counts of *IL8*, *CTGF*, *CXCL1*, *IL32*, *CYR61*, *CTHRC1*, *IL1A*, *IL1B* and *CEMIP* in CEMIP depleted cells (sh*CEMIP* #1 and #2) compared to non-depleted cells (shEGFP) . p-value adjusted (p-Adj) and Fold change (FC) between sh*CEMIP* (#1 or #2) and shEGFP from differential expression analyses performed with DESeq2.

Supplementary data 2

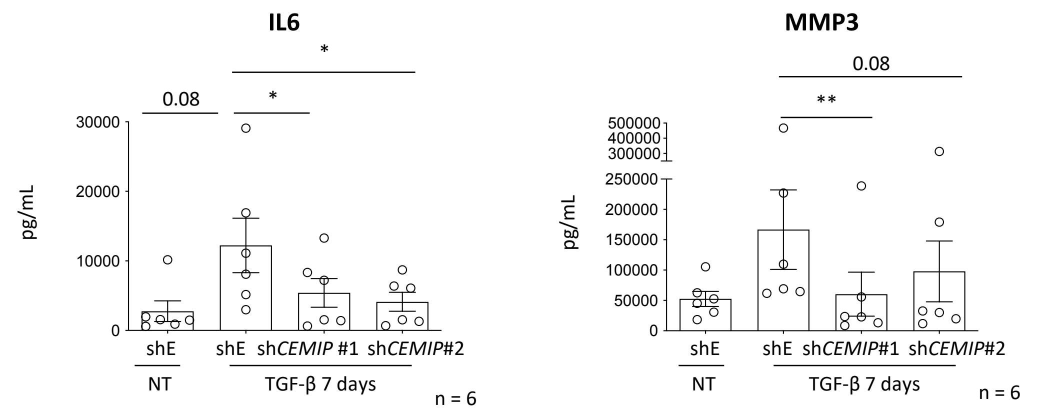

CEMIP regulates inflammation induced by TGF-β

ELISA analysis of IL-6 (left) and MMP-3 (right) secretion in *CEMIP* depleted cells (sh*CEMIP* #1 and #2) stimulated with TGF-β for 7 days compared to non-depleted cells (shEGFP) stimulated or not with TGF-β for 7 days (n=6).

Supplementary data 3

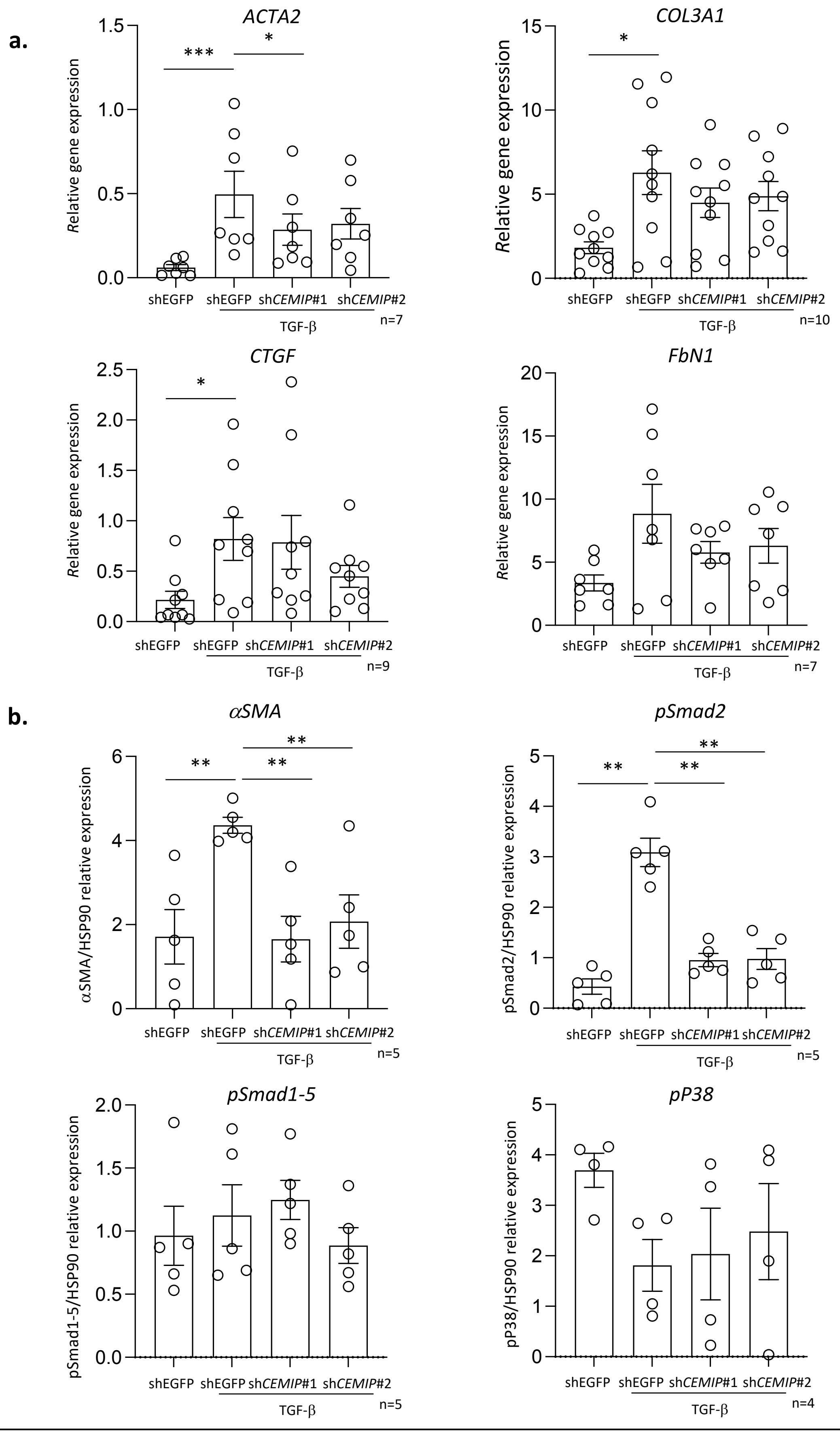

Regulation of fibrosis markers and TGF-β signaling by Cemip after 1 day of TGF-β treatment

RT-qPCR analysis of *ACTA2 (n=7), COL3A1 (n=10), CTGF (n=9) and FbN1 (n=7)* genes in CEMIP-silenced FLS (shCEMIP#1 and shCEMIP#2) compared to non- silenced cells (shEGFP) treated or not with TGF-β for 1 days (a). Western blot quantifications of aSMA (n=5), pSmad2 (n=5), pSmad1/5 (n=5) and p-p38 (n=4) in CEMIP-silenced cells (shCEMIP#1 and shCEMIP#2) compared to non-silenced cells (shEGFP) treated or not with TGF-β for 1 days.

Supplementary Data 4

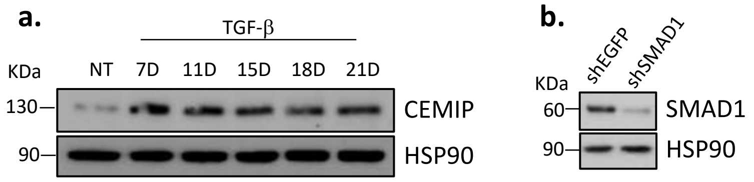

TGF-β stimulation induces CEMIP expression
Representative western blot analysis of CEMIP expression after TGF-β stimulation during, 7, 11, 15, 18 and 21 days (a). Representative western blot analysis of SMAD1 expression after treatment with shEGFP and shSMAD1 (b).
